# Three-dimensional mapping of mechanical activation patterns, contractile dyssynchrony and dyscoordination by two-dimensional strain echocardiography: Rationale and design of a novel software toolbox

**DOI:** 10.1186/1476-7120-6-22

**Published:** 2008-05-30

**Authors:** Bart WL De Boeck, Borut Kirn, Arco J Teske, Ralph W Hummeling, Pieter A Doevendans, Maarten J Cramer, Frits W Prinzen

**Affiliations:** 1 Department of Cardiology, University Medical Centre Utrecht, Heidelberglaan 100, 3584 CX Utrecht, The Netherlands; 2 Department of Physiology, University of Maastricht, Cardiovascular Research Institute Maastricht, Maastricht University, PO Box 616, 6200 MD Maastricht, The Netherlands; 3 Protys BV, Radex Innovation Centre, Rotterdamseweg 183c, 2629 HD Delft, The Netherlands

## Abstract

**Background:**

Dyssynchrony of myocardial deformation is usually described in terms of variability only (e.g. standard deviations SD's). A description in terms of the spatio-temporal distribution pattern (vector-analysis) of dyssynchrony or by indices estimating its impact by expressing dyscoordination of shortening in relation to the global ventricular shortening may be preferential. Strain echocardiography by speckle tracking is a new non-invasive, albeit 2-D imaging modality to study myocardial deformation.

**Methods:**

A post-processing toolbox was designed to incorporate local, speckle tracking-derived deformation data into a 36 segment 3-D model of the left ventricle. Global left ventricular shortening, standard deviations and vectors of timing of shortening were calculated. The impact of dyssynchrony was estimated by comparing the end-systolic values with either early peak values only (early shortening reserve ESR) or with all peak values (virtual shortening reserve VSR), and by the internal strain fraction (ISF) expressing dyscoordination as the fraction of deformation lost internally due to simultaneous shortening and stretching. These dyssynchrony parameters were compared in 8 volunteers (NL), 8 patients with Wolff-Parkinson-White syndrome (WPW), and 7 patients before (LBBB) and after cardiac resynchronization therapy (CRT).

**Results:**

Dyssynchrony indices merely based on variability failed to detect differences between WPW and NL and failed to demonstrate the effect of CRT. Only the 3-D vector of onset of shortening could distinguish WPW from NL, while at peak shortening and by VSR, ESR and ISF no differences were found. All tested dyssynchrony parameters yielded higher values in LBBB compared to both NL and WPW. CRT reduced the spatial divergence of shortening (both vector magnitude and direction), and improved global ventricular shortening along with reductions in ESR and dyscoordination of shortening expressed by ISF.

**Conclusion:**

Incorporation of local 2-D echocardiographic deformation data into a 3-D model by dedicated software allows a comprehensive analysis of spatio-temporal distribution patterns of myocardial dyssynchrony, of the global left ventricular deformation and of newer indices that may better reflect myocardial dyscoordination and/or impaired ventricular contractile efficiency. The potential value of such an analysis is highlighted in two dyssynchronous pathologies that impose particular challenges to deformation imaging.

## Background

The deleterious effects of an altered electrical activation on ventricular mechanical function have been recognized for the first time some 40 years ago but have gained important scientific interest only in more recent years [1]. Since, it has become clear that important disparities exist between electrical dyssynchrony and its mechanical consequences. The physiology behind these disparities is complex; it encompasses non-linear relationships between electrical and mechanical activation times [2-4] and involves an intricate interplay between loco-regional differences in wall stress, workload and contractility [5-8]. Electrical dyssynchrony can thereby induce a variable degree of unbalanced myocardial forces. Spatial differences in forces provoke spatial heterogeneities in timing and amplitude of myocardial deformation and also give way to segmental interactions within the heart (back-and-forth shortening and stretching between different regions) [7,9-11]. By this mechanism, part of total deformation work is dissipated into internal interaction work instead of being externalized into stroke work. Multiple well controlled studies have indicated that it is this heterogeneity of wall stress and deformation that determines both the functional impairment as well as the remodelling observed in the dyssynchronous ventricle [2,6-8,10,12-14]. Moreover, the benefits of cardiac resynchronization therapy (CRT) have been shown to be directly proportional to the reduction in deformation heterogeneity and dyscoordination [3,14,15]. Finally, the spatial organization of dyssynchrony – random versus organized – has been suggested to determine the chances of successful resynchronization and its pattern is considered important in choosing the most appropriate pacing site [3,15]. Therefore, myocardial deformation plays a pivotal role in the physiology of dyssynchrony and resynchronization. Nevertheless, a considerable gap persists between the experimental knowledge obtained from animal experiments and the complex physiology of dyssynchrony and response to therapy in human pathologies. Hence, for a proper evaluation of dyssynchrony in human subjects, both regional and global deformation have to be assessed by accurate techniques and with appropriate analysis methods. In the present work, we describe a novel software toolbox designed to improve the echocardiographic assessment of the above mentioned physiological aspects of dyssynchrony of deformation, we illustrate how this can provide new data in two challenging patient groups, and we discuss potential advantages and limitations of different approaches to quantify dyssynchrony.

## Materials and methods

### Patients and volunteers

Healthy controls (NL; n = 8) were included after providing written informed consent if they had a normal resting electrocardiogram and no cardiovascular disease and medication. Eight patients with Wolff-Parkinson-White syndrome (WPW), admitted for radio-frequency ablation of the accessory pathway underwent echocardiography the day before the procedure to rule out underlying structural abnormalities. All provided written informed consent. Seven patients with drug-refractory NYHA-class III heart failure (EF 18.8 ± 4.8%), widened QRS (179 ± 28 ms) and left bundle branch block (LBBB), of which 3 with an ischemic aetiology, underwent an extensive echocardiographic examination as part of the routine clinical work-up, an average of 45 ± 49 days before CRT. The exam was repeated before discharge, 2.5 ± 2 days after device implantation. The execution of the study conformed with the local Medical Ethics Committee policy and with the principles outlined in the Declaration of Helsinki on research in human subjects.

### Echocardiography acquisition

Echocardiography was performed on a GE Vingmed Vivid 7 scanner (GE Vingmed Ultrasound, Horten, Norway). Small angle, single wall, B-mode recordings of the septal, anteroseptal, anterior, lateral, posterior and inferior wall were performed from 3 standard apical imaging planes at 51 to 109 frames per second [16]. From the Doppler recordings of mitral inflow and left ventricular outflow the duration of the RR-interval, the timing of mitral valve opening (MVO) and closure (MVC), the onset of atrial flow wave (AWO), and aortic valve opening (AVO) and closure (AVC) were measured with respect to the onset of the QRS to serve as "reference timing events". Two-dimensional longitudinal and transverse strain and strain-rate curves were processed off-line using commercially available speckle-tracking software (GE, EchoPAC version 6.0.1). For each wall six samples were evenly distributed from base to apex, providing a 36-segment model of the left ventricle. Spatial smoothing was set at half of the software default value and the onset of the ECG was taken as the zero reference point. The obtained traces were transferred as text-files to a personal computer for post-processing into custom-made software (STOUT: Speckle tracking Toolbox Utrecht) programmed in Matlab (The MathWorks Inc., Natick, USA). The exported text-files contained information on the wall under investigation within the filename and the EchoPac software automatically generated a header to the numeric data encoding the type of parameter (velocity, strain, strain-rate, etc), its direction (longitudinal, transverse, etc.), the time of the zero reference point at onset and end of the cycle (defining the R-R-interval), and a colour-coding for the six levels.

### Data post-processing in STOUT

**Figure 1:**
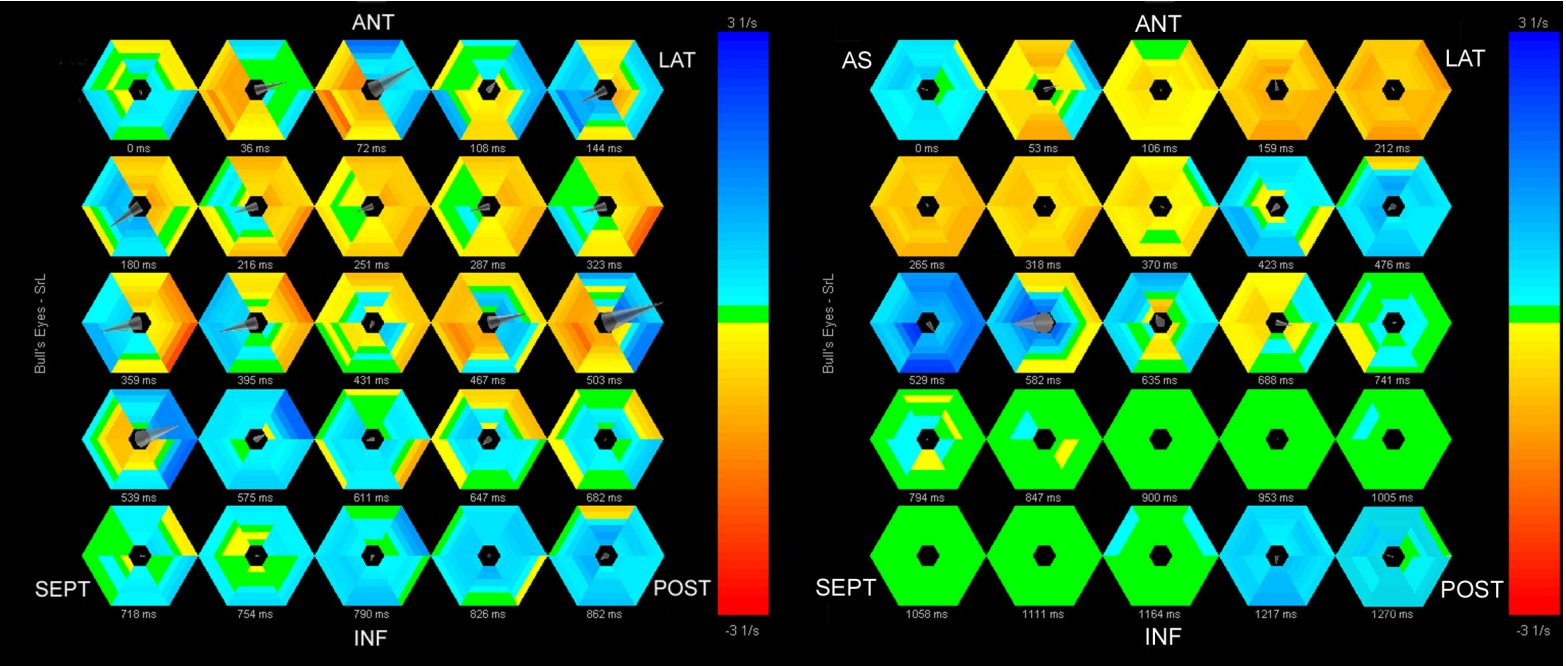
**Shortening and stretching patterns in a patient with LBBB (left) and a normal individual (right) represented by a series of colour-coded bulls-eyes representing deformation-rate at 25 time points throughout the entire cardiac cycle for each of the 36 segments.** Yellow = shortening, blue = stretching, green = no deformation/diastasis. AS = anteroseptum, ANT = anterior, LAT = lateral, POST = posterior, INF = inferior, SEPT = septum. In the normal individual (right), throughout most of the cardiac cycle all segments deform in phase (indicated by the same colour in all segments per frame). In LBBB (left) at many time points simultaneously occurring back-and-forth shortening (yellow) and stretching (blue) between septal regions and ventricular free wall can be appreciated.

**Figure 2:**
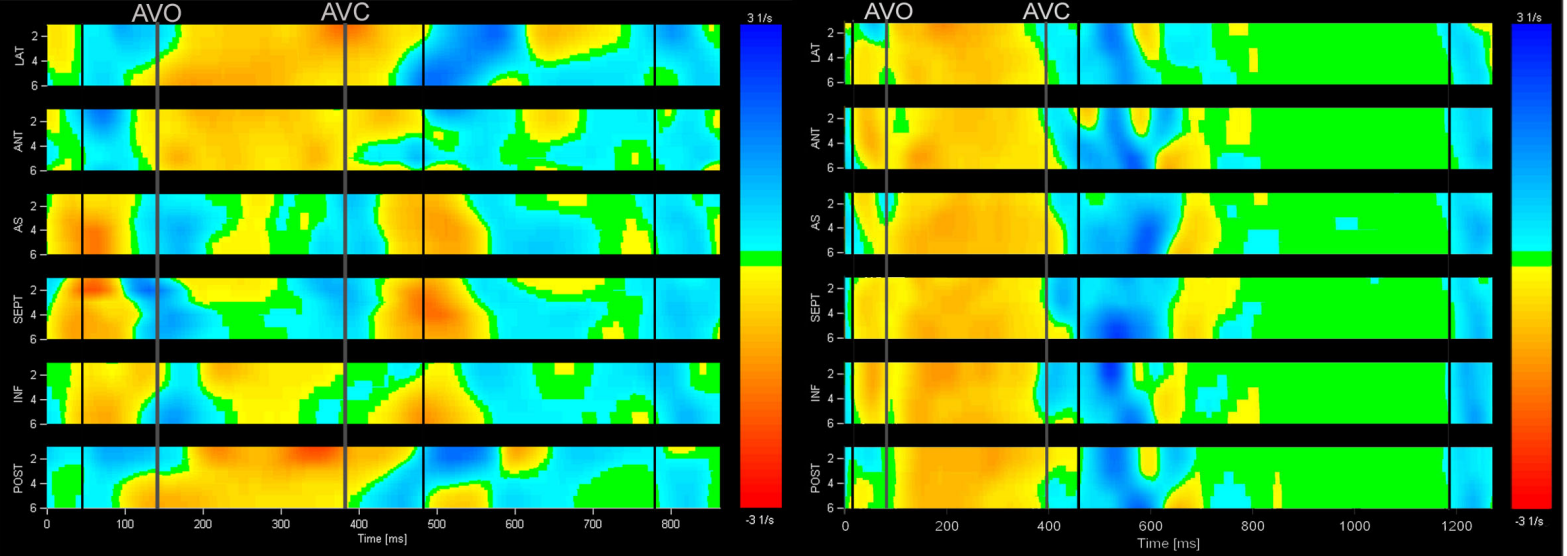
**Shortening and stretching patterns in a patient with LBBB (left) and a normal individual (right) represented by a two-dimensional M-mode map representation of the same data as in figure 1: the temporal information can now be continuously plotted over time (left to right within each plot).** Vertical lines represent event timing markers. Spatial representation is less optimal: each of the 6 walls is plotted separately (from top to bottom) with each level plotted from base (top) to apex (bottom) within the separate plots. As in figure 1, shortening and stretching are markedly inhomogeneous in LBBB (left) compared to NL (right).

**Figure 3:**
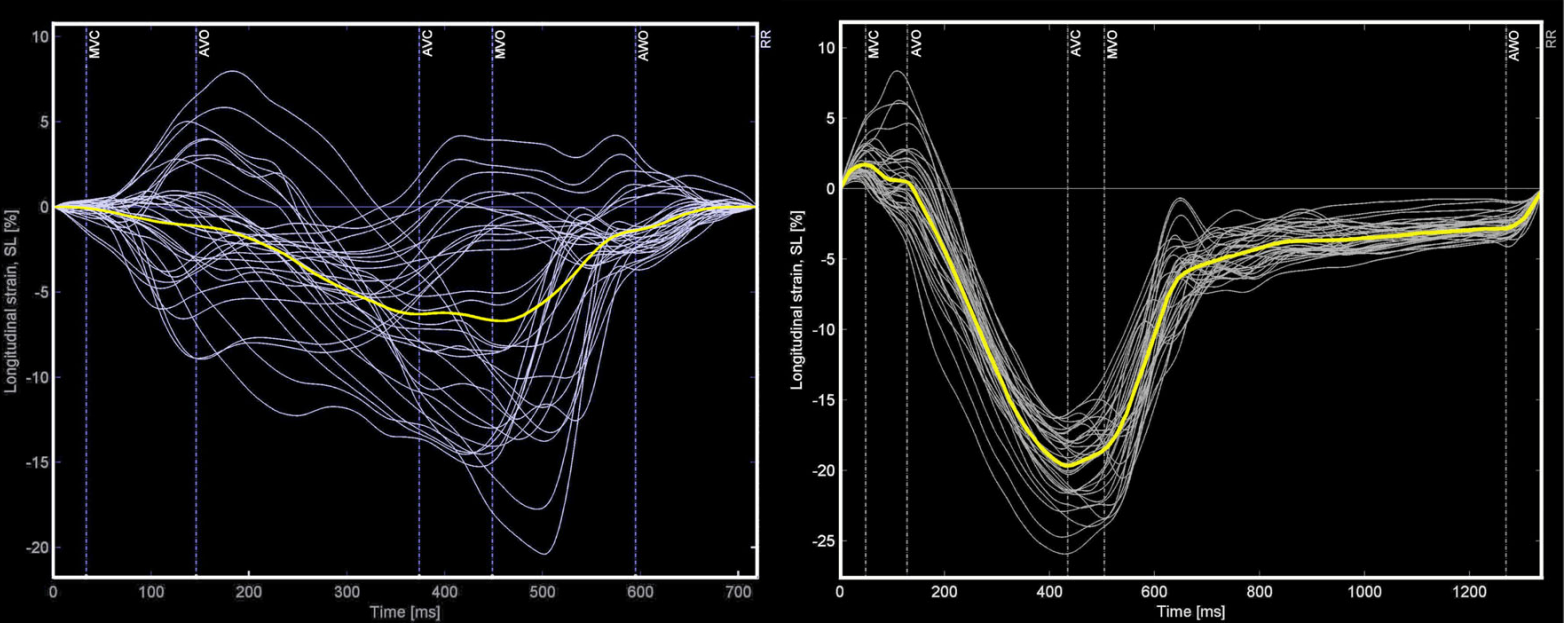
**Global strain plot in a patient with LBBB (left) and in a normal individual.** All 36 separate tracings are displayed as well as the average shortening curve representing global ventricular longitudinal shortening (thick yellow curve). Timing event markers are indicated in vertical dashed lines with MVC: mitral valve closure, AVO: aortic valve opening, AVC: aortic valve closure, MVO: mitral valve opening, and AWO: onset of mitral flow A-wave. Note the highly variable timing, shape and amplitude of the segmental deformation curves in this patient with LBBB (left) compared to the normal pattern (right).

In STOUT, the information embedded in the imported files regarding type of parameter, wall segment, beginning of the QRS and duration of the cardiac cycle is automatically decoded file by file. The "reference timing events" are imported manually once, after which adjustment for unequal frame rates and RR-intervals is automatically performed within each imported file by interpolating the data to 1 ms and by re-sampling based on the empirical observation that the systolic period lengthens with about 33% when total RR duration doubles; [17] see Additional file 1: Algorithm for RR-normalization. These RR-normalized and interpolated data are consecutively fitted to a simple 36 segment 3-D model assuming all walls to have similar length and a rotational orientation of 60° between the imaging planes [18]. The integration of spatial and continuous temporal information permits to display the data as a series of bulls-eyes (figure 1) and two-dimensional M-mode maps (figure 2), as well as a 4-dimensional projection of the data on a conical cast. By normalizing the individual curves to the reference RR, all data can be summed and averaged to yield a "global" or "netto" curve, representing the externalized motion or deformation of the ventricle as far as the dataset is complete (figure 3).

To make the analysis more time efficient, STOUT has an automated search algorithm for the identification of onsets, peaks and end-systolic values of motion and deformation. All data can be manually edited if needed. The vector algorithm proposed by Zwanenburg et al., allowing estimation of 3-D vectors also in case of missing values, is implemented in the software and is automatically calculated for the operator-approved onsets and peaks [19]. At the end of the analysis, all the results are automatically exported to an excel-spreadsheet.

**Figure 4:**
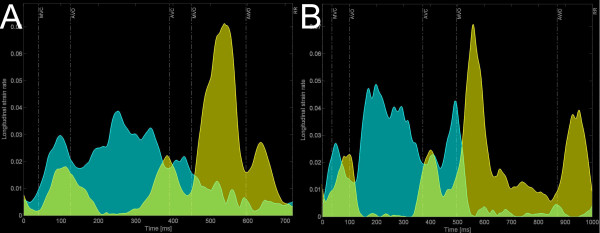
**ISF-plot with timing markers in a patient before (A) and after (B) CRT.** To calculate ISF (in this article between AVO and AVC), all shortening (in blue) and all lengthening strain-rates (in yellow) are summed and integrated over the desired time period. Overlapping areas (light green) indicate simultaneous shortening and stretching between different segments (dyssynergy). A/Note large areas of overlap between AVO and AVC in this patient with LBBB, resulting in a high ISF. B/Immediately after CRT, ISF has decreased (less overlap) because little lengthening occurs during ejection and the amount of shortening (blue area) has increased. This suggests conversion of internal strain into external (global) strain.

A new index estimating the impact of segmental interaction and expressing dyssynergy (i.e. dyscoordination/opposing strain work) is implemented in the software. The calculation of the internal strain fraction (ISF) is based on the directional changes of the strain, i.e. the slopes of the all strain curves, which for each time span are ranked in a group of shortening and lengthening/thickening strain slopes [20]. The absolute values of all these slopes for that particular time span (the actual value of the strain-rate) are summed within the two groups and plotted over time as a positive strain-rate group and a negative strain-rate group which are integrated over time to yield total positive and total negative strain (figure 4). ISF represents their relative fraction for the desired period within the cardiac cycle; see Additional file 2: Algorithm for ISF and vector of paradoxical strain-rate behavior (PSrV).

### Definitions and data analysis

Mechanical activation time was defined as the time of onset of shortening and throughout the article we will use mechanical activation for the onset of shortening [10]. The *temporal variability *of mechanical activation was then expressed by the standard deviation of shortening onset times (SDot) and its *spatio-temporal distribution width *by the vector magnitude of onset times in the horizontal plane (VMot). In analogy, the temporal variability (= standard deviation) and the spatio-temporal distribution width (= vector magnitude) were also calculated for the time to the (first) peak of shortening (SDpt and VMpt, respectively). These indices express dyssynchrony based on timing-issues only. The coefficient of variation of end-systolic strains (CVeS) was calculated to express the effects of dyssynchrony in terms of heterogeneity in strain amplitudes at end-ejection [13]. The inefficiency caused by deviation of the peak shortening from its ideal timing at AVC was estimated by comparing the peak deformation values with the end-systolic values in two ways. According to the first approach, the impact of dyssynchrony on global ventricular function (= degree of inefficiency) was expressed by estimating the improvement in global end-systolic shortening if all peak shortening were to occur at end-systole. As this represents a virtual resynchronization towards AVC it was denominated the virtual shortening reserve (VSR).

VSR = [(mean of peak strains - mean of end-systolic strains)/mean peak strains]*100%

**Figure 5:**
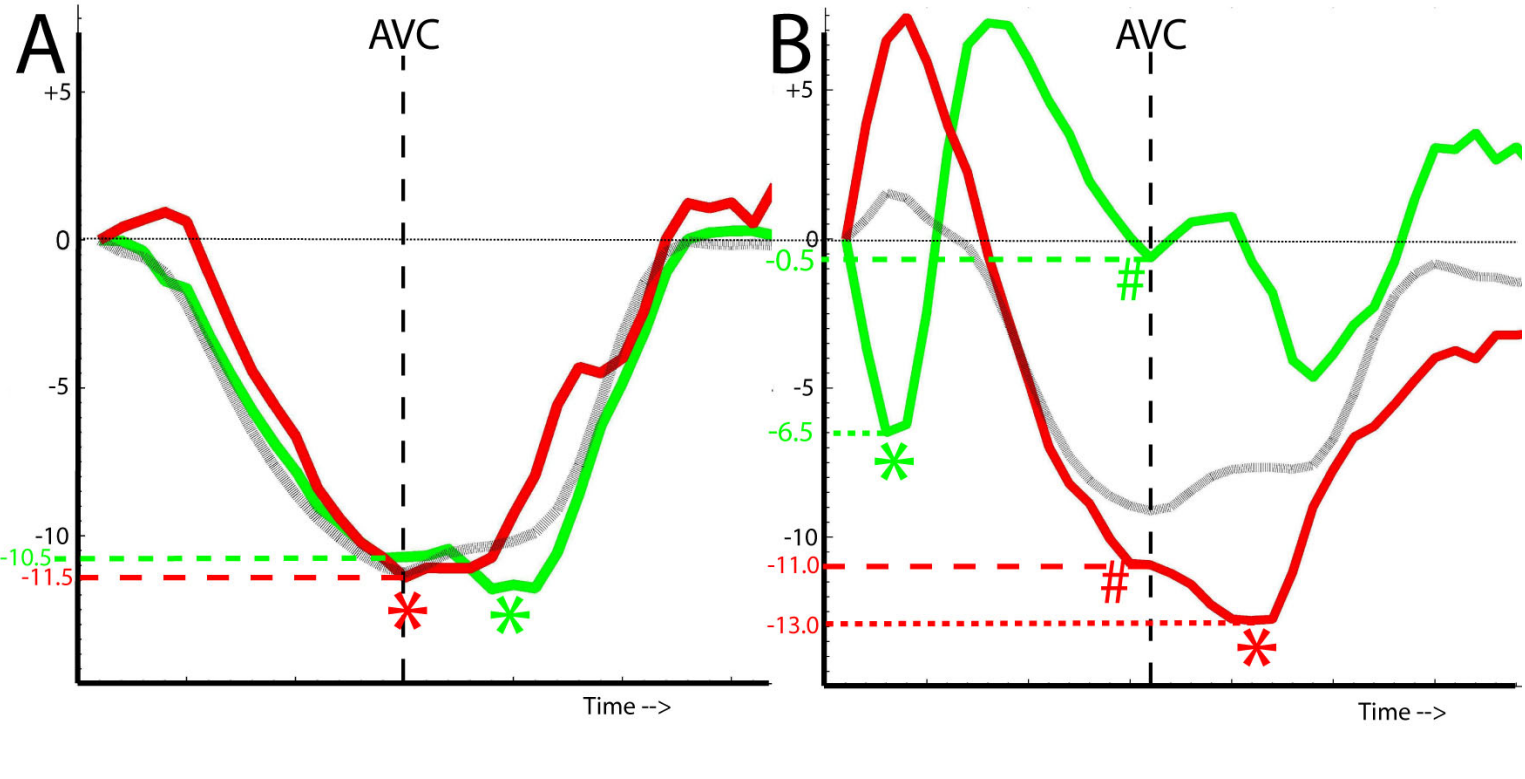
**Rationale for the use of ESR.** Representative deformation traces of the septal (green) and lateral wall (red) and global ventricular deformation (light grey) obtained by MR-tagging before (A) and 8 weeks after the induction of left bundle branch block (B) in a dog with on Y-axis shortening in % (data from reference 14). AVC = time of aortic valve closure; # and dashed line from AVC to Y-axis denote the end-systolic value, * denotes the peak value of deformation. From A to B: LBBB induces a marked reduction in septal strain peak amplitude (green *) and in particular in the end-systolic value (green #). The peak deformation amplitude of the lateral wall (red *) occurs after AVC and has increased (-11.0% to -13%) but the end-systolic value has changed less. This means that a hypothetically perfect resynchronization (backwards from B to A) would consist of an relative increase in septal deformation with little change in the lateral contribution in this period. Hence, to estimate how much function can improve by resynchronization, ESR only takes differences between peak and end-systolic values of early shortening into account (i.c.green* and green #).

For the second approach, the distinction was made between premature (peak shortening before AVC) and postsystolic shortening (peak at or after AVC). Only the inefficiency caused by premature shortening was considered to be amenable by resynchronization, while post-systolic shortening was not considered to represent recruitable shortening (figure 5). Hence the early shortening reserve (ESR) was used to estimate the amount of potentially amenable dyssynchrony by performing a virtual resynchronization of early shortening only: ESR = [(mean of premature peaks + mean of end-systolic strains of postsystolic peaks) - mean end-systolic value of all peaks)/(mean of peaks of early strains + mean of end-systolic strains of late peaks)]*100

The internal strain fraction (ISF) was used to express the impact of segmental interaction on ventricular function. ISF was calculated for the ejection period, defined in this study as the time between AVO and AVC. To describe the global left ventricular shortening during ejection, the global ejecting strain (GejS) was determined automatically from the internal strain rate plot:

GejS = (|total positive strain| - |total negative strain|) between AVO and AVC.

The site of earliest electrical activation was searched for by looking for the site of earliest mechanical activation within the ventricle was as well as on the origin of the vector of mechanical activation time by an observer blinded to the electrophysiological procedure (figure 4). In patients with WPW-syndrome, we compared the results with the localization of the bundle defined by electrophysiological mapping [21] and in patients following CRT with left ventricular lead position (all V-V ≤ 0 ms).

### Statistical analysis

Data between NL, WPW and LBBB at baseline were compared by ANOVA with Bonferroni correction for multiple comparisons. Dyssynchrony of mechanical activation (SDot, VMot) was compared to the corresponding dyssynchrony of peak deformation (SDpt, VMpt) by paired t-test in each patient group. The effect of CRT was also studied by comparison of pre-CRT values (LBBB) with post-CRT values (CRT) by paired samples T-test. A p-value of < 0.05 was considered statistically significant. Agreement between first mechanically activated site and localization of the extra bundle (WPW) or left ventricular lead (BiV) was described for circumferential segment.

## Results

Post-processing of the longitudinal deformation data imported in STOUT required 5 minutes on average for calculation and checking/editing of SDot, SDpt, VMot, VMpt and CVeS and for calculation and plotting of ISF.

### Variability and spatio-temporal distribution of mechanical activation and peak deformation

**Figure 6:**
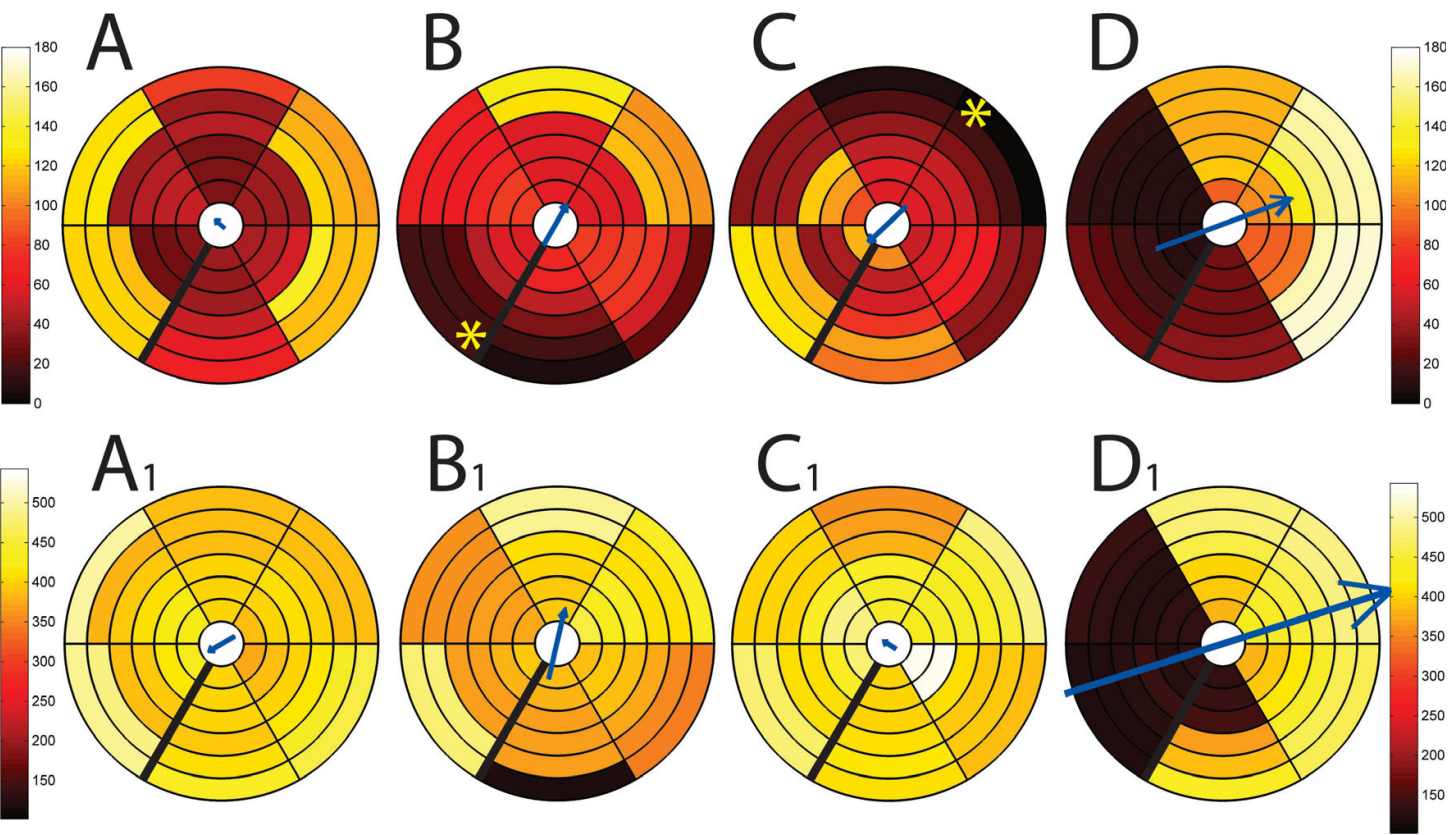
**Colour-coded bulls-eye projections of mechanical activation times (top row; A to D) and of timing of peak shortening (bottom row; A_1_-D_1_), with the vector magnitude and direction plotted in the centre (small blue arrows).** The thick black line on the left of each bulls-eye defines the attachment of the inferior right ventricular wall between the inferior and the septal wall (wall segmentation: see figure 1). For clarity, all plots have an equal scale: 0 ms to 180 ms for mechanical activation times (top row) and 100 to 550 ms for peak times (bottom row). Example A and A_1_: normal volunteer. Examples B, B_1 _and C, C_1_: patient with an inferoseptal and with an anterolateral bypass, respectively (* = invasively determined bypass localization). D: patient with LBBB. Note the large onset delay vector (D) and even larger peak shortening vector (D_1_) pointing from the septum to the lateral wall.

Table 1 displays the differences in timing-based dyssynchrony indices of longitudinal strain. Failing hearts with LBBB were characterized by a markedly increased temporal variability (SDot, SDpt) as well as spatio-temporal distribution width of deformation (VMot, VMpt) compared to the normal ventricles. Moreover, all these parameters in LBBB yielded significantly higher values at peak shortening compared to the onset of shortening. To the contrary, the distinction between WPW and NL could only be made by VMot, which was significantly larger in WPW, while dyssynchrony values at peak shortening were little different from the onset value in both groups (Table 1, figure 6).

**Table 1:** Timing based differences in deformation (mean ± SD) and Bonferroni-corrected p-value between groups.

**Group**	**SDot (ms)**	**SDpt (ms)**	**VMot (ms)**	**VMpt (ms)**
**NL**	42.2 ± 10.1	39.6 ± 10.3	28.7 ± 12.5	47.3 ± 27.8
**WPW**	37.9 ± 7.3	51.2 ± 12.8	77.1 ± 39.1 *	92.9 ± 35.2
**LBBB**	71.3 ± 9.0 #	135.2 ± 36.9 # ‡	143.4 ± 48.0 #	303.2 ± 109.9# ‡

# p < 0.001 versus NL and versus WPW; * p = 0.041 WPW versus NL; ‡ p < 0.01 versus SDot and VMot (paired t)

SDot: standard deviation of onset times; SDpt: standard deviation of peak times; VMot: vector magnitude of onset time distribution; VMpt: vector magnitude of peak time distribution.

### Indices of (the impact of) deformation heterogeneity and/or dyssynergy

**Figure 7:**
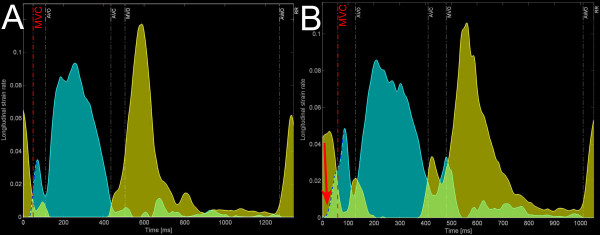
**Comparison of the ISF-plot obtained in a normal volunteer (A) and in a patient with WPW (B).** Note the increased area of overlap (light green areas) around the isovolumic periods (between MVC-AVO and between AVC-MVO) and in particular the onset of vigourous shortening (onset of high blue spike, red arrow) starting during the end of atrial filling, long before MVC (red dashed line).

Table 2 shows the baseline differences in CVeS, VSR, ESR and ISF. All parameters of deformation heterogeneity (CVES, VSR, ESR) and dyssynergy (ISF) were highly abnormal in LBBB, compared to NL as well as to WPW. None of the parameters reached statistical difference when comparing NL and WPW. Observation of the ISF-plots identified pre-excitation induced dyssynergy of deformation shortly after the QRS and mostly before AVO rather than during ventricular ejection in WPW, compatible with local abnormal early shortening during late passive filling and isovolumic contraction (figure 7 and Additional file 3: figure showing PSrV plot in NL and WPW).

**Table 2:** Markers of deformation heterogeneity and dyssynergy (mean +/- SD) and Bonferroni-corrected p-value. CVeS: coefficient of variation of end-systolic strain values; VSR: virtual shortening reserve; ESR: early shortening reserve; ISF: internal strain fraction during the ejection period.

**Group**	**CVeS (%)**	**VSR (%)**	**ESR (%)**	**ISF (%)**
**NL**	-16.4 ± 4.4	4.8 ± 2.4	1.8 ± 2.2	3.9 ± 1.8
**WPW**	-24.3 ± 7.1	8.4 ± 3.5	3.4 ± 3.5	6.2 ± 3.2
**LBBB**	-124.4 ± 60.5 #	46.3 ± 13.3 #	37.0 ± 15.0 #	47.9 ± 19.1 #

CVeS: coefficient of variation of end-systolic strain values; VSR: virtual shortening reserve; ESR: early shortening reserve; ISF: internal strain fraction during the ejection period.

### Effects of resynchronization

In this small sample of patients, a resynchronization effect of biventricular pacing was not demonstrable when dyssynchrony was expressed merely in terms of variability of deformation timing (SDot, SDpt) or end-systolic amplitudes (CVeS). However, biventricular pacing markedly diminished VMot, VMpt, indicating a decreased spatial divergence of deformation timing (Table 3). A more detailed analysis of the vector data also indicated that biventricular pacing with a mean V-V-interval of -43 ± 37 ms, had inverted the septal to lateral mechanical activation delay vector to a small lateral to septal delay (121.8 ± 50.0 ms to -30 ± 44.8 ms, p < 0.0001). Analysis by ISF suggested that the overall coordination of shortening had improved and this improved coordination during the ejection was paralleled by an improvement in ejection performance (GejS) (Table 3).

**Table 3:** Effects of CRT on homogenization (SDot, SDpt) and redistribution (VMot, VMpt) of timing of deformation and on parameters expressing deformation heterogeneity (CVeS, VSR, ESR), synergy (ISF) and global function (GejS and ejection fraction).

Parameter	pre-CRT (LBBB)	post-CRT (CRT)	p-value
SDot (ms)	71.3 ± 9.0	56.9 ± 16.9	0.050
SDpt (ms)	135.2 ± 36.9	124.3 ± 28.5	0.100
VMot (ms)	143.4 ± 48.0	79.3 ± 40.5	**0.049**
VMpt (ms)	303.2 ± 109.9	125.4 ± 82.6	**0.003**
CVeS (%)	-124.4 ± 60.5	-173.3 ± 246.0	0.540
VSR (%)	46.3 ± 13.3	36.4 ± 22.1	0.124
ESR (%)	37.0 ± 15.0	19.2 ± 16.0	**0.005**
ISF (%)	47.9 ± 19.1	22.2 ± 9.6	**0.006**
GejS (%)	-3.5 ± 1.4	-6.0 ± 2.6	**0.029**
Ejection fraction (%)	18.8 ± 4.8	24.2 ± 6.0	**0.018**

### Site of ventricular pre-excitation in WPW and LV-first pacing

The presence of a bypass with electrical pre-excitation somewhere at the ventricular base instead of around the apical anterior and septal breakthrough site in the normal ventricle inverted the mechanical activation vector from an apex to base (apex-base vector component NL: 38 ± 30 ms) pattern to a base to apex mechanical activation gradient (apex-base vector component WPW: -19 ± 21 ms, p = 0.015). The origin of the mechanical activation vector in the horizontal plane was variable and correctly indicated the site of the bundle in 7 out of 8 patients (figure 6). The site of earliest mechanical activation matched with the site of the bypass in 6 of the 8 patients. The same methodology identified the site of the left ventricular pacing in 5 out of 7 cases (both for vector and earliest mechanical activation). In all cases of incorrect echocardiographic diagnosis, the site of electrical pre-excitation was located in the adjacent segment.

## Discussion

In the current work, we illustrate how two-dimensional deformation data obtained from echocardiography can be reconstructed into a 3-D model of the left ventricle in order to enable a more comprehensive description of mechanical activation and deformation dyssynchrony. Such an approach enables mapping of the spatio-temporal distribution characteristics of dyssynchrony and allows the implementation of newer indices aimed at estimating the impact of dyssynchrony on global ventricular performance. Using a model of mild dyssynchrony (WPW), severe dyssynchrony (LBBB) and an intervention on the dyssynchronous substrate (CRT), differences between and potential advantages of certain approaches are further discussed.

### Differences between approaches to express dyssynchrony and dyscoordination

The most widely used method to describe dyssynchrony consists of measuring differences in timing of onsets and/or peaks of deformation throughout the ventricle [10,22,23]. However, multiple shortening waves are very common in the dyssynchronous ventricle, making this method vulnerable to noise and rendering uniform definitions on "onsets" and "peaks" more cumbersome [10,22,23]. When spatial information is encoded in the data-set, delays within the ventricle can also be expressed in terms of their spatio-temporal distribution patterns, e.g. by vector-analysis [10,19]. This approach may be preferable as it offers additional data on the organizational pattern of deformation and makes the analysis less vulnerable to accidental outliers or random noise in the measurements. In the present study, the additional value of vector analysis became apparent in the WPW-ventricle. A distinctly different spatio-temporal pattern of mechanical activation compared to the normal ventricle could be demonstrated, while in this small group no differences were detectable in variability of mechanical activation. Moreover, in the patients with left bundle branch block, CRT had a stronger effect on the vector magnitude than on the standard deviation of peak shortening timing [10].

Myocardial dyssynchrony does not only induce heterogeneity of deformation-timing but also of -amplitudes. Because global ventricular function relates to global deformation, [24,25] and global deformation in turn depends on the deformation magnitude in the individual wall segments as well as on the coordination (synergy) between them, timing alone does not necessarily reflect the impact of the disturbance. Nelson et al. used CVeS as a marker of dyssynchrony in patients with idiopathic dilated cardiomyopathy and demonstrated that CVeS strongly predicted the acute benefit of CRT [13]. However, not only dyssynchrony but also regional ischemia or scarring can affect end-systolic strain variance [26]. Accordingly, in a recent study involving ischemic and non-ischemic patients, this parameter seemed less valuable [27].

VSR represents a novel way to estimate the impact of dyssynchrony on global function. By "weighing" the observed end-systolic strain to the peak strain it may somewhat compensate for the aforementioned shortcoming of CVeS. Indeed, the VSR-value is insensitive to timely peaking but hypokinetic contractile behaviour. Nevertheless, in the present study neither CVeS nor VSR significantly changed upon resynchronization (see next paragraph: rationale for ESR).

ISF is a another new approach to estimate the impact of dyssynchrony by reflecting the part of the total deformation that is lost internally due to *simultaneous *shortening and stretching because of dyssynchrony [20]. ISF is rather a dyssynergy (= dyscoordination) than a dyssynchrony marker since it regards "synchrony of contraction" as "simultaneous shorten*ing *or lengthen*ing *in all parts of the ventricle". When different wall segments are deforming in phase with each other, there's synergy and ISF will be zero regardless of differences in velocity and extent of deformation. However, when some wall segments are deforming out of phase, the velocity and extent of their abnormal deformation do determine ISF. Hence, ISF becomes independent of the choice of peaks while remaining sensitive to strain amplitude differences of dyssynchronous segments. Preliminary results with ISF of circumferential shortening obtained by MR-T suggest this index of segmental interaction to be better related with long term remodelling than timing parameters only [20]. Of interest, the present study indicates that CRT improves global ventricular function (GejS, ejection fraction) by a reduction of ISF, i.e. by a conversion of internal into external shortening. The fact that spatial distribution patterns cannot be deducted from ISF and that its value can be affected by random noise may represent limitations; with a small adaptation of the algorithm however, 3-D vectors of out-of-phase or paradoxical strain behaviour can be calculated throughout the cardiac cycle (See Additional file 2: Algorithm for ISF and vector of paradoxical strain-rate behavior (PSrV) and Additional file 3: Additional figure showing PSrV plot in NL and WPW). This fell beyond the scope of the present work.

The advantages of the proposed method in comparison with other techniques are summarized in the table attached in the appendix of this document (Additional file 4: Table 4: Comparison of commonly used echocardiographic techniques/indices to evaluate mechanical dyssynchrony with STOUT-indices)

### Dyssynchrony analysis by myocardial deformation: unmet challenges

A key issue in the treatment of mechanical dyssynchrony is that electrical therapies-like CRT – can only amend electrical dyssynchrony [28]. Unfortunately, heterogeneity of deformation and mechanical dyssynchrony are not always caused by electrical dyssynchrony [29]. An imbalance in active and passive forces, causing deformation heterogeneity, can also occur in the absence of electrical activation delays [26]. One of the true challenges for deformation imaging therefore lies in the distinction between mechanical dyssynchrony based on electrical dyssynchrony or based on other local conditions [26,30,31]. In (local) pathologies such as ischemia for example, delayed and post-systolic shortening is rather a passive phenomenon of recoil than an expression of amendable dyssynchrony and premature shortening may represent a more specific marker. In addition, the relative amplitude changes in premature and delayed segments seen in animal experiments of acutely induced left bundle branch block suggest that postsystolic shortening in general may not represent shortening that can be recruited towards the end of the ejection period (see figure 5). Excluding post-systolic shortening from the analysis by confining measurements to the ejection period (e.g. by ISF) or by disregarding postsystolic peaks as in the calculation of ESR may therefore improve the estimation of truly recoverable dyssynchrony. In accordance with the latter hypothesis, VSR was not significantly changed by CRT in the present study, while ESR was significantly reduced.

### Modelling of deformation: comparison with previous work

Myocardial deformation or strain can reliably be measured in vivo by magnetic resonance tagging (MR-T) imaging [24,32]. This technique has been applied in animal models also during CRT, instigating the development of highly effective therapies like cardiac resynchronization therapy (CRT) [3,7,10,14,15]. However, in humans MR-T has practical constraints and not all human's pathology can accurately be represented in animal models. Strain echocardiography by speckle tracking is a valuable alternative [16,33]. However, particularly in spherically dilated, thin walled and hypokinetic ventricles, the temporal and spatial resolution of echocardiography and the signal to noise ratio of speckle tracking are challenged. Frame rate, focus position and sector width can be adapted to optimize the ultrasound beam density and image quality in order to improve the reliability of speckle tracking [16]. We therefore designed the current software in such a way that segmental data from single wall recordings can be imported separately if needed.

It has been recognized previously that the assumptions and algorithms used for data interpolation and incorporation into a 3-D model can alleviate but also introduce sources of error [18]. However, all current deformation imaging modalities depend on reconstruction techniques and all are particularly vulnerable to grossly irregular heart rates. Because exact spatial location, orientation and geometry are known when MR-T is used, true 3-dimensional MR-T data sets can be reconstructed. Nor with the current, nor with a previously proposed echocardiographic methodology this is possible [18]. With 3-D based speckle tracking software soon becoming available, the latter problem might be solved in the near future. Nevertheless, and in spite of using longitudinal instead of circumferential deformation, our ISF, dispersion, and vector data on mechanical activation and dyssynchrony closely resemble the published MR-T data in normal individuals and in patients with LBBB [13,19,20,34].

Echocardiographic strain analysis can be applied also in humans with contraindication to MR-T, such as following CRT. This has offered unique data on the effects of CRT in humans in the current and in previous studies [35]. Finally, 2-DSE can measure deformation throughout the entire cardiac cycle and is thus independent of QRS triggering or fading of the taglines in diastole. This offers new opportunities. In the current work this is illustrated by providing the first preliminary data on mechanical activation vectors and dyssynchrony in WPW-patients.

### Limitations

The presented echocardiographic approach remains time consuming and laborious, in particular related to the care taken to obtain high quality single wall recordings, the need for a meticulous registration of the timing events and the subsequent off-line calculation of deformation by the EchoPac-software at each of the wall segments individually. Once all files are transferred to STOUT however, little extra time is spent at the actual analysis of the traces and at making the dyssynchrony results available for statistical analysis. Another drawback of the methodology is that many of the presented indices will offer valuable information only when image quality is sufficient to provide robust deformation results covering most of the ventricle. In clinical practice, this can be problematic even when attempting to optimize quality by a single wall approach. The presented image acquisition and post-processing approach might therefore better serve research purposes than clinical practice but we expect newly gained insight to generate simpler methods for routine practice. One of such clinically more feasible methods to predict response to CRT for example, might be the calculation of ESR deducted from the septum only, as previous and the present work indicates that in LBBB the septal segments generally are the earliest (vector of peak time), display most stretching towards end-systole [3,7,10,14,15] and thereby likely contribute most to the ESR-value.

Myocardial deformation is a complex three-dimensional event and differences in synchrony and synergy between the main axes of deformation have been suggested [36]. In the present study we only reported on longitudinal deformation parameters. Transverse data from the same long-axis images and at the same location can be processed in STOUT, as well as circumferential, radial and transverse data. Although this allows a direct comparison, such study fell beyond the scope of the present work.

In the present study we primarily intended to highlight the differences (in strength) between the individual dyssynchrony indices and to point out some physiological aspects that have to be taken into consideration when expressing dyssynchrony and dyscoordination. Only a limited number of patients were therefore included in the present study. It is important to recognize that in recent literature many new technologies and dyssynchrony indices have been put forward, in some cases without providing either the pathophysiological rationale for their use nor a standardized methodology. In particular in the field of cardiac resynchronization therapy, many of them have entered the clinical arena long before being properly evaluated in multi-centre trials against simpler and user-friendly methods. Each new method should therefore be scrutinized regarding its rationale and tested for its feasibility and reliability in the real world. This is no different for the currently proposed indices; whether the higher sensitivity of a vector-, ISF- and/or ESR-based approach found in this study translates into a superior clinical yield remains to be established in larger, prospective studies.

## Conclusion

Ample experimental data and sound physiologic principles support the use of deformation imaging in the study of the nature and the impact of mechanical dyssynchrony. A dedicated software toolbox was designed to reconstruct myocardial deformation data obtained by 2-D speckle tracking echocardiography into a simple 3-D model of global ventricular deformation. This allowed the calculation of 3-D vectors of mechanical activation and of global left ventricular deformation. The software was also designed to allow the implementation of newer indices better reflecting important pathophysiological aspects of myocardial dyscoordination and impaired ventricular contractile efficiency. A comprehensive description of the spatio-temporal characteristics and of the impact of dyssynchrony of myocardial deformation by echocardiography might prove helpful in particular in pathologies in which magnetic resonance imaging has practical constraints, such as WPW and following CRT.

## Competing interests

The authors declare that they have no competing interests.

## Authors' contributions

BDB conceived the study, designed the software, acquired and analysed part of the data and drafted the manuscript. BK participated in the study design and conceived the index ISF. AT acquired part of the study data and was involved in drafting the manuscript. RH wrote the software, formatted and implemented the new algorithms into Matlab. PD and MC critically revised the article for important intellectual content. FP made substantial contributions to the conception of the study and the interpretation of data, critically revised the article for important intellectual content and helped to draft the manuscript. All authors have read and approved the final manuscript.

## Supplementary Material

Additional file 1 Algorithm 1: RR-normalization. The file describes the algorithm implemented in Matlab to time-normalize different deformation curves to a single, common reference RR-interval. Click here for file

Additional file 2 Algorithm 2: Internal Strain Fraction and vector of paradoxical strain-rate behavior (PSrV). The file describes the basic principle and practical implementation of two novel indices of dyscoordination, ISF and PSrV, into STOUT. Click here for file

Additional file 3 Example of a PSrV plot in a normal individual and a patient with WPW. The plot displays the vector magnitude of paradoxical strain-rate in the horizontal plane at 20 ms time-steps. Paradoxical deformation behaviour will increase the magnitude (and R^2 ^of the estimation) of the vector when it is more vigorous, when it encompasses a more extensive area or both, unless it is due to random noise. Unreliable vectors (R^2 ^< 0.40) have been omitted. The presence of a highly reliable and large PSrV before mitral valve closure (MVO) is seen only in the WPW-ventricle (arrow), indicating vigorous shortening during late atrial filling with a well organized spatial pattern. This corresponds to premature shortening in the pre-excited area while in other area's stretching from the atrial contraction is ongoing. Click here for file

Additional file 4 Comparison of commonly used echocardiographic techniques/indices to evaluate mechanical dyssynchrony with STOUT-indices. A table outlining the main theoretical and technical advantages of a STOUT-based approach over more conventional echocardiographic techniques to study dyssynchrony. Click here for file
