# Supplementary material for: Three-dimensional mapping of mechanical activation patterns, contractile dyssynchrony and dyscoordination by two-dimensional strain echocardiography: Rationale and design of a novel software toolbox
Source: Cardiovasc Ultrasound. 2008 May 30;6:22. doi: 10.1186/1476-7120-6-22 (PMC2429897; doi:10.1186/1476-7120-6-22)
Supplement: Additional file 1 — Algorithm 1: RR-normalization. The file describes the algorithm implemented in Matlab to time-normalize different deformation curves to a single, common reference RR-interval. [file 1476-7120-6-22-S1.doc]

**Algorithm 1: RR-normalization**.

Definitions: 1/ Time span onset first QRS (t=0) to onset next QRS = RR

2/ “systole” = time span t=0 to mitral valve opening (MVO)

Assumptions: A/ Time between onset of A-wave (AWO) and RR varies insignificantly (RR-AWO is fixed)

B/ empirically: if RR*2, then “systole”*1.33

Implementation:

Each curve (i) can be divided in 3 time-intervals:
  from t=0 to MVO (i) ->  d1(i)

from MVO (i) to AWO (i) ->  d2(i)
  from AWO (i) to RR (i) ->  d3(i)

*After curve-normalization to the reference RR*, these time-intervals are known: RR, MVO and AWO are the reference timing markers obtained by Doppler at the reference RR- interval at which Doppler was performed.

All curves(i) are normalized in such a way that they will match these reference timing markers.

For each curve (i) MVO(i), AWO(i) and R-R(i)are determined and consecutively all other time-values of the curve are fitted to the following equation:

f·RR(i) = RR
f·RR(i) = f1·d1(i) + f2·d2(i) + f3·d3(i)

R-R(i) is known (encoded in the imported file information), hence

f = R-R / R-R(i)
  
For each time interval, a scaling factor is determined that fulfills the following requirements:

  f1·d1(i) = d1
  f2·d2(i) = d2
  f3·d3(i) = d3
 
Because RR - AWO is fixed, f3 = 1, so d3(i) = d3 and AWO(i) = RR(i) - d3

The analytical relation between f1 and f reads (assumption B):

  f1 = 5/3 log(8f)/log(32)  (e.g.for f=1, f1=1; for f=2,f1=1.33)

and MVO(i) = MVO / f1

With MVO(i) and AWO(i)known,

  f2 = d2 / d2(i) => d2 / (AWO(i) - MVO(i))

The timevector (i) is now divided in the 3 known intervals d1(i), d2(i) en d3(i) multiplied with respectively f1, f2 en f3.

Finally, spline interpolation of the curves to 1 ms is performed over this new time vector.
