# Supplementary material for: Three-dimensional mapping of mechanical activation patterns, contractile dyssynchrony and dyscoordination by two-dimensional strain echocardiography: Rationale and design of a novel software toolbox
Source: Cardiovasc Ultrasound. 2008 May 30;6:22. doi: 10.1186/1476-7120-6-22 (PMC2429897; doi:10.1186/1476-7120-6-22)
Supplement: Additional file 2 — Algorithm 2: Internal Strain Fraction and vector of paradoxical strain-rate behavior (PSrV). The file describes the basic principle and practical implementation of two novel indices of dyscoordination, ISF and PSrV, into STOUT. [file 1476-7120-6-22-S2.doc]

**Algorithm 2: Internal Strain Fraction and vector of paradoxical strain-rate behavior (PSrV)**.

Definitions: Deformation or strain = ε

Slope of the strain curve = strain-rate = ∆ε

Principle: **ISF:** Calculations based on the slope of the strain-curves, i.e. ∆ε, which are ranked in a group of shortening / thinning (-) and lengthening / thickening (+) strain slopes at each time span and thereafter summed within the group over the desired time period.

**PSrV:** Calculation based on ∆ε of local curves y, which are compared to the ∆ε of the global ventricular deformation curve for each time span of 20 ms. All curves in which ∆ε has similar polarity as the global ∆ε have “0” assigned as value, all others get ‌‌│∆ε │assigned. On these values 3-D vectors are calculated (per 20 ms) pointing towards the largest / most vigorous out of phase deformation-rate and plotted over the R-R.

Implementation: **A: Internal strain fraction per period M**

Step 1: Ranking the ∆ε of each curve at timespan i into either the + (P∆ε) group or the – (N∆ε) group.

P∆ε_i_ = 1/ (2N) ***** $\sum_{k=1}^{N} (\left|\Delta\varepsilon i,k\right|-\Delta\varepsilon i,k)$

in which i = timespan unity, k = curve and N number of curves present in the analyses

N∆ε_i_ = 1/ (2N) ***** $\sum_{k=1}^{N} (\left|\Delta\varepsilon i,k\right|+\Delta\varepsilon i,k)$

Step 2: Integration of the groups over time (if M = QRS-onset to AVC: “systolic”)

(if M = AVO to AVC: “ejection”)

Total P∆ε_i_  over predefined time span M = $\sum_{i=1}^{M} \text{P}\Delta\varepsilon i$

Total N∆ε_i_  over predefined timespan M = $\sum_{i=1}^{M} \text{N}\Delta\varepsilon i$

Step 3: ISF-calculation

🡺 100*(Total P∆ε_i_ / Total N∆ε_i_ ) if Total N∆ε_i_ > Total P∆ε_i_ or

🡺 100*(Total N∆ε_i_ / Total P∆ε_i_ ) if Total N∆ε_i_ < Total P∆ε_i_

**B. Calculation of PSr-value at each segment, per time span i = 20 ms**

Step 1: Assigning either │∆ε i,y│ or 0 as paradoxical strain-rate value (PSr-value) for each curve at location y over time period i.

PSr-value i,y = 1/2$\left[sign\left(\Delta\varepsilon i,y\right)-sign\left(\Delta\varepsilon i,m\right)\right]*\Delta\varepsilon i,y$

In which ∆ε i,m the slope of the global strain curve

Step 2: Introducing of all PSr-values i,y to i,n in vector algorithm, plot of magnitude per 20 ms
