# Supplementary figures and images for: Three-dimensional mapping of mechanical activation patterns, contractile dyssynchrony and dyscoordination by two-dimensional strain echocardiography: Rationale and design of a novel software toolbox
Source: Cardiovasc Ultrasound. 2008 May 30;6:22. doi: 10.1186/1476-7120-6-22 (PMC2429897; doi:10.1186/1476-7120-6-22)

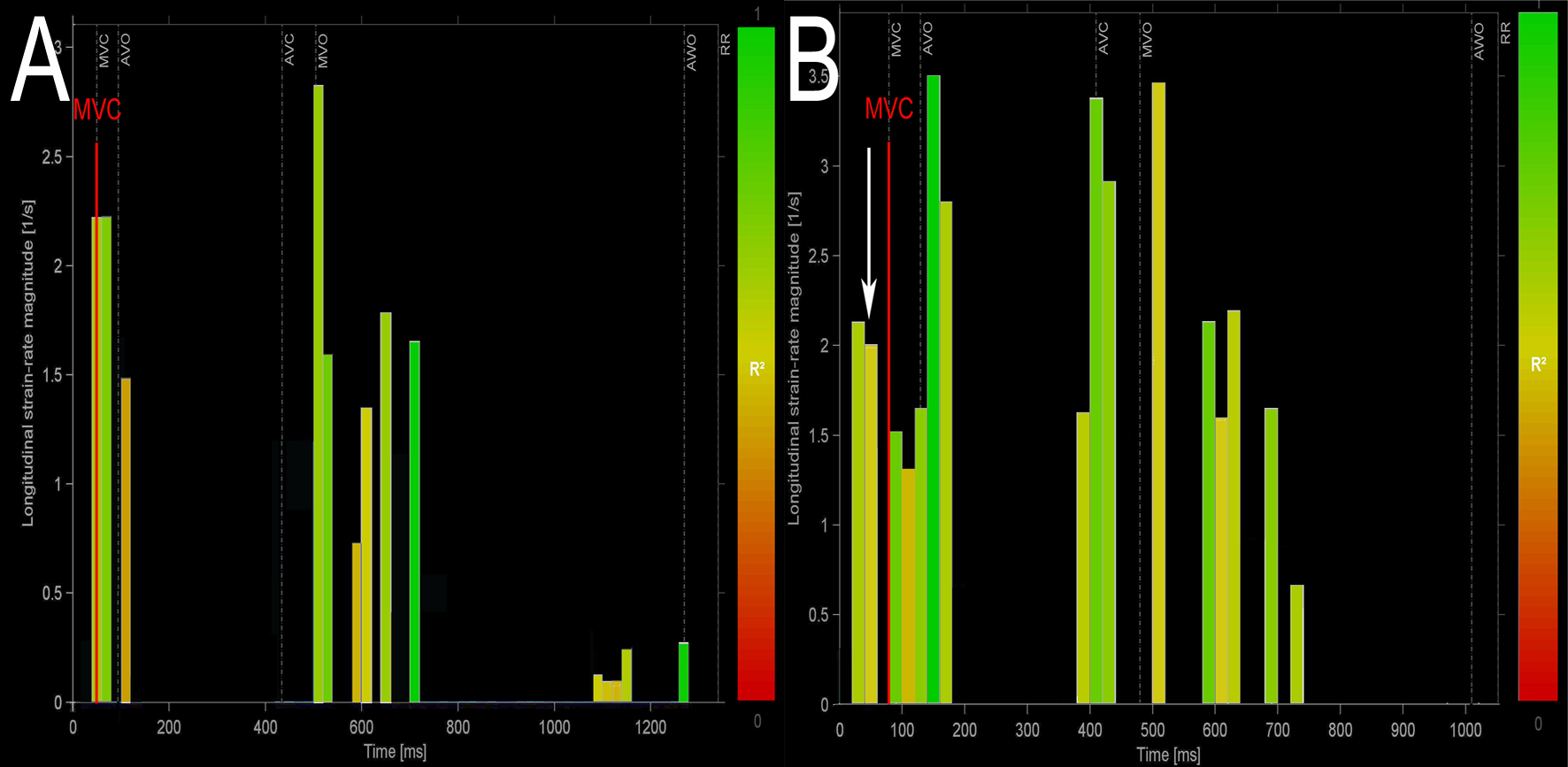

Supplement: Additional file 3 — Example of a PSrV plot in a normal individual and a patient with WPW. The plot displays the vector magnitude of paradoxical strain-rate in the horizontal plane at 20 ms time-steps. Paradoxical deformation behaviour will increase the magnitude (and R2 of the estimation) of the vector when it is more vigorous, when it encompasses a more extensive area or both, unless it is due to random noise. Unreliable vectors (R2 < 0.40) have been omitted. The presence of a highly reliable and large PSrV before mitral valve closure (MVO) is seen only in the WPW-ventricle (arrow), indicating vigorous shortening during late atrial filling with a well organized spatial pattern. This corresponds to premature shortening in the pre-excited area while in other area's stretching from the atrial contraction is ongoing. [file 1476-7120-6-22-S3.tiff]
