# Supplementary material for: Three-dimensional mapping of mechanical activation patterns, contractile dyssynchrony and dyscoordination by two-dimensional strain echocardiography: Rationale and design of a novel software toolbox
Source: Cardiovasc Ultrasound. 2008 May 30;6:22. doi: 10.1186/1476-7120-6-22 (PMC2429897; doi:10.1186/1476-7120-6-22)
Supplement: Additional file 4 — Comparison of commonly used echocardiographic techniques/indices to evaluate mechanical dyssynchrony with STOUT-indices. A table outlining the main theoretical and technical advantages of a STOUT-based approach over more conventional echocardiographic techniques to study dyssynchrony. [file 1476-7120-6-22-S4.doc]

Comparison of commonly used echocardiographic techniques / indices to evaluate mechanical dyssynchrony with STOUT- indices.

| **Technique**  strenght | Evaluation of dyssynchrony | | | | |
| --- | --- | --- | --- | --- | --- |
|  | Index is physiologic marker of | Spatiotemporal info / mapping | Temporal resolution | Estimator of functional impact | Operator independency |
| **Current technologies** |  | | | | |
| TDI /TSI (timing peaks) | ?? | +/- (1🡪2D/3D) | <10 ms | - | +/- |
| TDI-strain (timing peaks) | Timing local shortening / ejection | +/- (1🡪2D) | <10 ms (<5*) | +/- | - |
| 2-DSE (timing peaks) | Timing local shortening / ejection | + (2D) | < 20 ms | +/- | +/- |
| 3-D (timing min. volumes) | Timing local ejection | + (3D) | 35-50 ms | +/- | +(+) |
| **STOUT-reconstruction** |  | | | | |
| Vector on 2-DSE timing | Spatial sequence LV ejection | +(+) (2🡪3D) | 10-15 ms | +/- | + |
| ISF | LV shortening/ejection inefficiency | - | N.A. | +(+) | + |
| PSrV | Spatial organization LV inefficiency | + (2🡪3D) | per 20 ms | ? | +(+) |
| ESR | Inefficiency (amendable fraction?) | - | N.A. | +(+) | + |
| 2-D M-mode / bulls-eyes | Visual map spatiotemporal shortening | +(+) | <10-25 ms | - | +(+) |

Legend:

Techniques: TDI: Tissue Doppler (velocity) imaging; TSI: Tissue Synchronization imaging, automated measurement of peak velocities in 6 basal and 6 mid-ventricular segments; TDI-strain: TDI-derived strain imaging; (*) = if single wall acquisition. 2-DSE: Speckle-derived 2-D strain imaging as routinely applied on 2-, 3- and 4-chamber sector scans; 3-D: Full volume 3-D echocardiography with automated detection of time to minimal volumes; STOUT-reconstruction: incorporation of segmental strains obtained from single wall acquisitions into STOUT; ISF: Internal Strain Fraction; PSrV: Paradoxical strain-rate vector; ESR: early shortening reserve, 2-D M-mode / bulls-eyes :graphical representations of amplitudes over time, interpolated to 1 ms (M-mode) or divided into RR/25 (ms) see text for details.

Values: (1🡪2D) indicates the use of an intrinsically (angle-dependent) 1-D technique enabling comparison in 2D (or 3D by TSI); (2🡪3D) indicates the use of an intrinsically (in plane angle-independent) 2-D technique reconstructed into a 3-D model. Scoring by – denotes poor; by +/- moderate or only partially related to; by + good; by +(+) better / specifically designed for. N.A. = Not applicable, the index has no / is little dependent on temporal characteristics. For operator independency, independency to insonation angle and limited manual interventions by automation / computerization of technique and measurement are considered beneficiary. See text for details.
